# Efficiency of substitutive renal therapy in the complex intensive care of multiple organ failure in patients with polytrauma

**DOI:** 10.1186/cc12367

**Published:** 2013-03-19

**Authors:** S Kravtsov, A Shatalin, V Agadzhanyan, D Skopintsev

**Affiliations:** 1 Federal State Budgetary Medical Prophylactic Institution 'Scientific Clinical Center of the Miners' Health Protection', Leninsk-Kuznetsky, Russia

## Introduction

This study was to evaluate the efficiency of the early start of intermittent substitutive renal therapy in patients with polytrauma complicated by multiple organ failure syndrome.

## Methods

Forty-two patients with polytrauma complicated by multiple organ failure syndrome were included in the study. The age of the patients was from 20 to 60 years (38.3 ± 1.6 years average). All patients were divided into two equal groups. In the control group (CG) the criteria for the start of the substitutive renal therapy were: hyperkalemia ≥6 mmol/l, plasma creatinine ≥280 μmol/l, diuresis ≤20 ml/hour. In the investigation group (IG) there were subtests to carry out the substitutive renal therapy, allowing one to start it in the earlier period of the multiple organ failure progression. These are increase of Na^+ ^>150 mmol/l, osmolarity >300 mOsm/l, elevation of the plasma toxicity according to the average molecule concentration ≥1.0, diuresis decrease ≤40 ml/hour. These were examined: lethality, quantity of the substitutive renal therapy procedures, mechanical lung ventilation duration (MLV), intensive care and hospital duration. The substitutive renal therapy was carried out by AK-200-Ultra apparatus (Gambro, Sweden). The statistical analysis was realized using Statistica 6.1 and the Mann-Whitney U test.

## Results

The average quantity of the substitutive renal therapy procedures in the CG was 13.4 ± 0.7, in the IG it was 8 ± 0.6 (*P *0.05). The recuperation of the renal excretory functions was on 19 ± 1 day in 12 patients of the CG, and on 11 ± 1.3 day in the IG, from the moment of substitutive renal therapy start (*P *0.05). Lethality in the CG was 43% (nine patients), and in the IG it was 29% (six patients, *P *0.05). The duration of the MLV in the CG and IG was 21 ± 1.2 days and 16 ± 1.2, respectively (*P *0.05). In the IG the duration of the ICU was lower by 23%, hospitality duration was lower by 17% (*P *0.05).

## Conclusion

The efficiency of the substitutive renal therapy depends directly on the hydroelectrolytic and metabolic changes and toxicosis degrees in the polytrauma complicated by multiple organ failure syndrome. The early start of the dialysis methods treatment allows one to achieve the earlier recuperation of the renal functions and to decrease the lethality level by 14%.

